# Association between mortality and frailty in emergency general surgery: a systematic review and meta-analysis

**DOI:** 10.1007/s00068-020-01578-9

**Published:** 2021-01-09

**Authors:** Christophe Alain Fehlmann, Dilan Patel, Jessica McCallum, Jeffrey Joseph Perry, Debra Eagles

**Affiliations:** 1 School of Epidemiology and Public Health, University of Ottawa, Ottawa, ON K1G 5Z3 Canada; 2 Department of Anaesthesiology, Clinical Pharmacology, Intensive Care and Emergency Medicine, Geneva University Hospitals, 1211 Geneva, Switzerland; 3 Emergency Medicine Research Group, Ottawa Hospital Research Institute, Ottawa, ON K1Y 4E9 Canada; 4 Department of Emergency Medicine, University of Ottawa, Ottawa, ON Canada

**Keywords:** Systematic review, Frailty, Clinical frailty scale, Emergency general surgery

## Abstract

**Purpose:**

The purpose of this review was to determine the association between frailty and mortality among adults ≥ 65 years old undergoing emergency general surgery (EGS).

**Methods:**

This systematic review followed the PRISMA guidelines (CRD42020172482 on PROSPERO). A search in MEDLINE, PubMed, EMBASE, Scopus, Web of Science, and the Cochrane Database of Systematic Reviews was conducted from inception to March 5, 2020. Studies with patients ≥ 65 years undergoing EGS were included. The primary exposure was frailty, measured using the Clinical Frailty Scale or the Modified Frailty Index. The primary outcome was 30-day mortality. Secondary outcomes were 90-day and 1-year mortality, length of stay, complications, change in level of care at discharge, and loss of independence. Two independent reviewers screened articles and extracted data. Risk of bias was assessed according to the Newcastle–Ottawa Scale and quality of evidence was assessed using the GRADE approach. A meta-analysis was performed for 30-day mortality using a random-effects model.

**Results:**

Our search yielded 847 articles and six cohort studies were included in the systematic review. There were 1289 patients, 283 being frail. The pooled OR from meta-analysis for frail compared to non-frail patients was 2.91 (95% CI 2.00, 4.23) for 30-day mortality. Frailty was associated with increased odds of all secondary outcomes.

**Conclusion:**

Frailty is significantly associated with worse outcomes after emergency general surgery in adults ≥ 65 years of age. The Clinical Frailty Scale could be used to improve preoperative risk assessment for patients and shared decision-making between patients and healthcare providers.

**Registration number:**

CRD42020172482 (PROSPERO).

**Supplementary Information:**

The online version contains supplementary material available at 10.1007/s00068-020-01578-9.

## Introduction

In 2050, approximately one-quarter of the population in western countries will be over the age of 65 [1]. The number of unscheduled emergency department visits by this population has increased by 30% during the last 10 years [2]. Aging populations have increased the number of older patients presenting for emergency surgery, with patients over 60 representing greater than 30% of all emergency general surgery cases [3]. Given the significant proportion of older patients in the population, it is important to determine the impact of older age on healthcare outcomes.

Overall, 11% of general surgery cases are emergency general surgeries. Compared to elective surgery, emergency general surgery is associated with a fivefold higher mortality rate and a threefold higher complication rate [4]. In older patients, improvements and advancements in anaesthesiologic care and surgical techniques resulted in a decrease in mortality and post-operative complications in recent years. However, this remains an important issue, as their risk of death after emergency laparotomy is more than twice than that of patients less than 70 years old [5]. The predictors of mortality in older patients who undergo emergency general surgery warrant further investigation.

Frailty can be defined as “a condition or syndrome which results from a multisystem reduction in reserve capacity to the extent that a number of physiological systems are close to, or past, the threshold of symptomatic clinical failure”[6]. More than 50 tools have been developed to measure frailty [7]. Several studies have shown that frailty is associated with poorer outcomes: in the emergency department, frail patients are at increased risk of death or complications for several pathologies, such as acute coronary syndrome, trauma, pneumonia, and acute cardiac failure [8–12]. Concerning surgery, frailty was also associated with mortality, complications, and length of stay, independent of the type of surgery [13–15]. To our knowledge, there is no prior systematic review specifically assessing the impact of frailty on mortality among older patients who undergo emergency general surgery.

## Objectives

The primary objective of this systematic review was to assess the association between frailty and 30-day mortality after emergency general surgery in patients aged ≥ 65 years. Our secondary objectives were to summarize the association between frailty and 90-day mortality, 1-year mortality, complications, hospital length of stay, change in level of care at discharge, and loss of independence at any time.

## Methods

This study was submitted to PROSPERO on March, 6th 2020 and registered on April, 28th 2020. The protocol was not published, but is available upon request. It was amended on March 7th (regarding the requirement for 80% of patients to meet inclusion criteria in mixed studies) and April 5th (major complications being Clavien-Dindo ≥ 3, i.e., complications requiring intervention, life-threatening complications requiring admission to intensive-care unit, death) [16]. We conducted this systematic review and meta-analysis according to the preferred reporting items for systematic reviews and meta-analyses (PRISMA) guidelines (Online Appendix I)[17].

### Eligibility criteria

We included English-only studies reporting human-only original research (randomized-controlled trials, prospective or retrospective comparatives cohorts, and case–control studies). We included studies examining adults ≥ 65 years of age who underwent emergency general surgery. The age criterion was a firm cut-off and all study subjects were required to be ≥ 65 years of age. Emergency general surgery was defined as any of the following procedures: appendectomy, cholecystectomy, laparotomy, lysis of adhesions, large bowel resection, small bowel resection, and peptic ulcer repairs, performed on a non-elective basis [18]. Studies were eligible if they reported stratified data for emergency general surgery or if 80% or more of the patients had emergency general surgery. Studies were included if frailty was measured by the Clinical Frailty Scale (CFS) or the Modified Frailty Index (mFI) [19, 20]. Frailty was studied as a dichotomous variable (frail versus non-frail); patients with a Clinical Frailty Scale ≥ 5 or a Modified Frailty Index ≥ 3/11 were considered as frail (Online Appendix II, S1 and S2). These cut-offs are most commonly used [19, 21].

The primary outcome was 30-day mortality, defined as death during the 30-day period following emergency general surgery. Secondary outcomes included 90-day and 1-year mortality, defined as death at any time during the 90-day period or 365 day period following emergency general surgery, respectively; hospital length of stay, defined as either (a) the number of days between admission and discharge or (b) the number of days between surgery and discharge; major post-operative complications at any time, defined as a Clavien–Dindo score of 3 to 5, compared to 0–2 (Online Appendix II, S3) [16]; an increase in level of care at discharge; and loss of independence at any time. We originally defined major complications as a Clavien–Dindo score of 3 or 4 [excluding 5 (death)]; however, since all included studies presented complications with a Clavien–Dindo score of 5, we re-defined this outcome to include death. Letters, editorials, review articles, case reports, and case series (≤ 10 patients) were excluded. We excluded studies with patients aged < 65, patients who were followed up for less than 30 days following the surgery, and if the scores from the Clinical Frailty Scale or Modified Frailty Index were not presented as an absolute value or a dichotomous variable with our pre-specified cut-offs.

### Information source and search strategy

Our literature search strategy was developed using medical subject headings (MeSH) and text words related to emergency general surgery and frailty. We searched MEDLINE, PubMed, EMBASE, Scopus, Web of Science, and the Cochrane Central Register of Controlled Trials from inception until March 5, 2020. We also scanned the reference lists of included studies and relevant reviews identified through the search. The search strategy was developed with a medical librarian. Search terms related to emergency, surgery, and frailty scores were included. Emergency terms included terms such as expedited OR urgent OR emerg*. Surgical terms included terms such as surgery OR laparotomy OR cholecystectomy OR colectomy OR hernia OR adhesion OR incision OR drainage. Frailty terms included terms such as frail*. The full search strategy can be found in online Appendix III.

### Study selection

The results of the literature search were uploaded to Covidence Software [22]. Titles and abstracts yielded by the search were independently screened by CF and another reviewer (DP or JM). Discrepancies were resolved by the third reviewer. Full-text reports meeting inclusion criteria were reviewed by CF and another reviewer (DP or JM). Discrepancies were resolved by the other reviewer. Duplicates were removed either electronically during the search or manually during screening. If two or more papers reported the results for the same outcomes in the same study, only the study with the larger sample size was selected. Authors were contacted if study data were not stratified by frailty scale or not stratified by surgery type to determine if they met eligibility criteria.

### Data extraction

A pre-designed, standardized data extraction sheet was created using Excel^©^. Two reviewers independently collected the pre-specified data. Disagreements were resolved by the third reviewer (DP or JM). For each study, we collected publication details (author, year of publication, country, journal), study details (study design, eligibility criteria, number of patients included, funding resource), type of frailty measure, and sample size of frail and non-frail. The pre-specified outcomes (including 30-day, 90-day, and 1-year mortality, complications, hospital length of stay, change in level of care at discharge, and loss of independence at any time) were extracted according to frail and non-frail for each group, in each study. Unadjusted and adjusted odds ratios were also collected. If essential data such as outcomes stratified by frailty scores, used for computing odds ratios, were not reported, study authors were contacted.

### Risk of bias in individual studies

Risk of bias was evaluated using the Newcastle–Ottawa Scale (NOS) for cohort studies [23]. For our review, bias was only assessed for the main outcome of interest that was extracted. If there was insufficient detail reported, we judged the risk of bias as ‘unclear’. Bias was evaluated independently by two review authors and disagreements were resolved by consulting the third reviewer (DP or JM).

### Data synthesis

Clinical heterogeneity was evaluated based on study population, design, and assessment of the outcomes. When at least two studies were judged to be sufficiently clinically homogeneous, a meta-analysis was conducted using a random-effects model. We pooled dichotomous data and reported odds ratios and 95% confidence intervals. Statistical heterogeneity was then evaluated through the *I*^2^ statistic. If this statistic was greater than 75%, we planned to explore possible sources of heterogeneity. When, for some outcomes, there were not enough data to effectuate a meta-analysis, results were reported descriptively. We planned to assess for potential publication bias by visual inspection of funnel plots. Review Manager 5.1 (Copenhagen: The Nordic Cochrane Centre, The Cochrane Collaboration, 2014) was used for all statistical analyses [24].

### Confidence in cumulative evidence

We planned to assess the quality of evidence for every outcome with a meta-analysis using the GRADE (grading of recommendations assessment, development, and evaluation) approach [25]. Only studies included in the meta-analysis were used for the assessment of the strength of evidence. Since a meta-analysis was only possible for the primary outcome, the GRADE approach was not used for secondary outcomes.

## Results

### Study selection

**Fig. 1:**
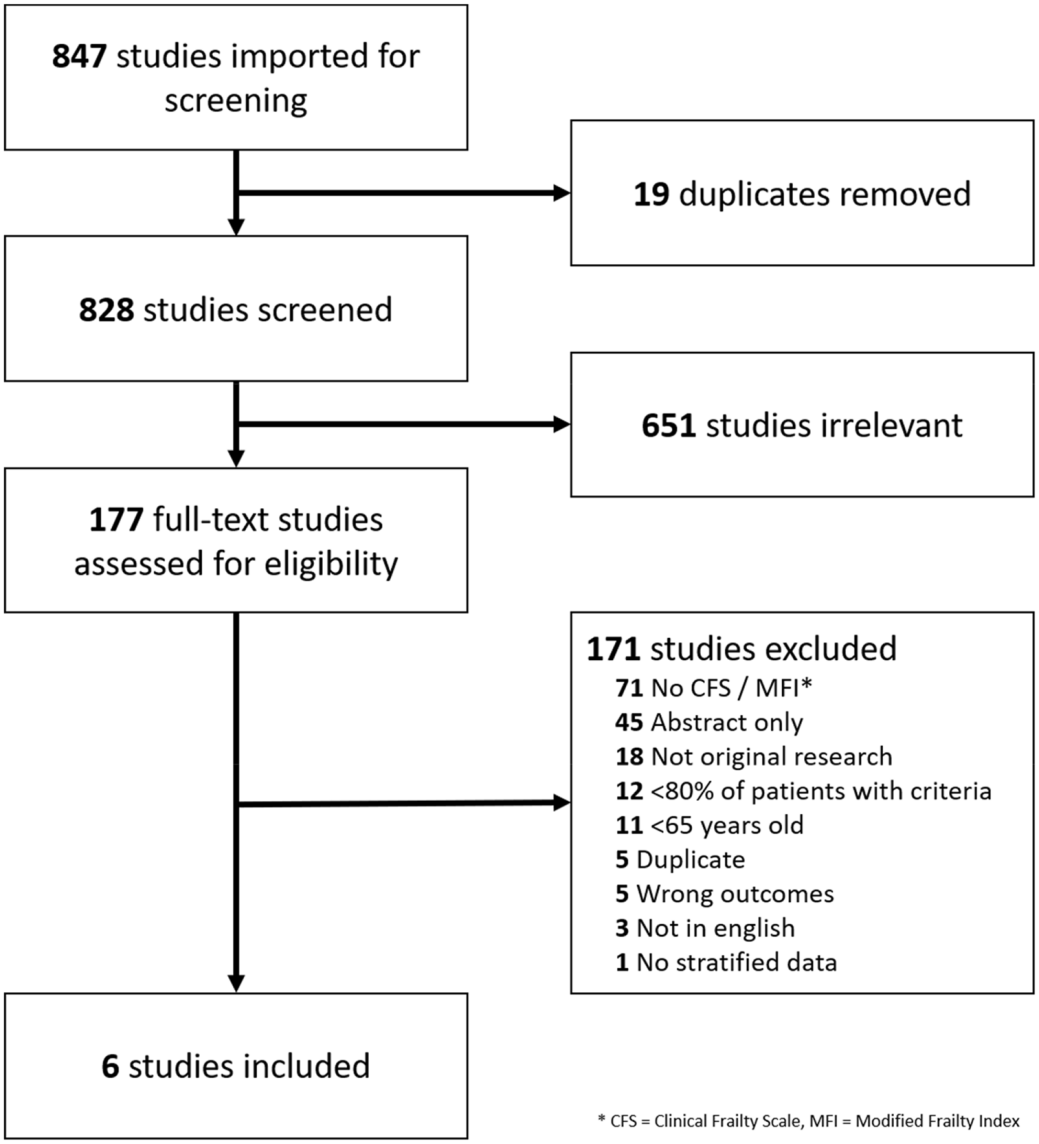
Flowchart of search strategy and studies selection

The search strategy identified 847 titles and abstracts, 19 duplicates were removed, 828 titles and abstracts were screened, and 651 studies were excluded yielding 177 full texts for review (Fig. 1). Six studies from five cohorts were included (five full studies and partial data from one study including colorectal and upper gastrointestinal surgery only) [26–31]. The main reason for the exclusion of full texts was if frailty was measured by alternative methods other than the Clinical Frailty Scale or Modified Frailty Index, or not measured at all.

### Study characteristics

Information of included studies is presented in Table 1. Five were prospective cohort studies and one was a retrospective cohort. Study patients were enrolled between June 2012 and April 2019. They were conducted in the United Kingdom [26, 28, 29, 31], Singapore [27], and Spain [30]. Five of them reported frailty measured by the Clinical Frailty Scale [26, 28–31] and only one by the Modified Frailty Index [27]. Inclusion criteria were 65 for four studies [26–29], and 70 and 75 for the two other studies [30, 31].

**Table 1:** Study characteristics of the included studies

Authors, year, and journal	Design	Country	Period	Frailty measure	Inclusion criteria	Exclusion criteria	Funding
McGuckin et al., Anaesthesia [26]	Retrospective Cohort	United Kingdom	June 2012 – January 2013	CFS	Age ≥ 65 Unscheduled non-cardiac surgery Stratified data: Colorectal and upper gastrointestinal surgery	None	Research institute
Tan et al., World Journal of Emergency Surgery [27]	Prospective Cohort	Singapore	June 2016–February 2018	MFI	Age ≥ 65 Emergency abdominal surgery (including diagnostic laparoscopies and emergency abdominal wall hernia repairs)	Vascular, gynaecological and transplant surgeries Emergency operations for complications of elective surgery Patients who were not expected to survive the index admission	University, Hospital
Parmar et al., Annals of Surgery[28]^a^	Prospective cohort	United Kingdom	20 March 2017–19 June 2017	CFS	Age ≥ 65 Expedited, urgent, or emergency surgical abdominal procedure for gastrointestinal pathology (laparoscopic or open procedure) Returning to theatre for any major postoperative complication/dehiscence	Diagnostic intervention Appendicectomy only Cholecystectomy only Vascular surgery, including abdominal aortic aneurysm repair Laparotomy/laparoscopy for pathology caused by blunt or penetrating trauma	Research foundation
Carter et al., British Journal of Surgery [29]^a^	Prospective cohort	United Kingdom	20 March 2017–19 June 2017	CFS	Age ≥ 65 Expedited, urgent, or emergency surgical abdominal procedure for gastrointestinal pathology (laparoscopic or open procedure) Returning to theatre for any major postoperative complication/dehiscence	Diagnostic intervention Appendicectomy only Cholecystectomy only Vascular surgery, including abdominal aortic aneurysm repair Laparotomy/laparoscopy for pathology caused by blunt or penetrating trauma	Research foundation
Arteaga et al., European Journal of Trauma and Emergency Surgery [30]	Prospective cohort	Spain	September 2017–April 2019	CFS	Age ≥ 70 Emergency abdominal surgery	Moderate-severe cognitive deterioration Terminal illness, defined as a life expectancy of less than 6 months	None
Vilches-Moraga et al., Aging Clinical and Experimental Research [31]	Prospective cohort	United Kingdom	September 2014–March 2017	CFS	Age ≥ 75 Emergency general surgery	Diagnostic intervention Appendicectomy only Cholecystectomy only Vascular surgery, including abdominal aortic aneurysm repair Laparotomy/laparoscopy for pathology caused by blunt or penetrating trauma	None

*MFI* Modified Frailty Index, *CFS* Clinical Frailty Scale

^a^For those studies, patients were part of the National Emergency Laparotomy Audit (NELA), which has specific inclusion and exclusion criteria. Only important criteria have been mentioned in the table. Moreover, the articles from Parmar and Carter report results from the same cohort

### Patient characteristics

Patient characteristics and outcomes are shown in Table 2. The six included studies were comprised of 1289 different patients (718 females, 283 frail patients). The smallest study sample size was 38 patients [26] (stratified data from a larger study) and the largest was 937 patients [28]. Half of the patients of each study were female, and the prevalence of frailty was between 20 and 32%. Clinical heterogeneity in reporting of demographic data in the studies precluded pooling of all other demographic variables of interest except gender.

**Table 2:** Patient demographics and relevant outcomes for included studies

Authors	Sample size	Frailty measure	Female *N* (%)	Frail *N* (%)	Relevant outcomes
McGuckin et al. [26]	38	CFS	18 (47)	11 (29)	*30-day mortality* Frail patients: 2/11 (18.2%) Non-frail patients: 1/27 (3.7%) Unadjusted OR = 5.78 *Length of stay* Frail patients: mean 54.2 days (SD = 77.3) Non-frail patients: mean 38.3 days (SD = 54.0)
Tan et al. [27]	109	MFI	51 (47)	22 (20)	*Loss of functional independence at 1 year* Compare to patients with MFI 1–2, patients with MFI ≥ 3 has an unadjusted OR 4.42 for the outcome *Complications* Frail patients: 1/22 (4.5%) Non-frail patients: 6/87 (6.9%) Unadjusted OR = 0.64 *Length of stay* Frail patients: mean 15.5 days (SD = 9.6) Non-frail patients: mean 14.3 days (SD = 9.7)
Parmar et al. [28]	937	CFS	540 (58)	190 (20)	*90-day mortality* Frail patients: 62/189 (32.8%) Non-frail patients:121/741 (16.3%) Unadjusted OR = 2.50 Compare to patients with CFS = 1, the adjusted ORs were 0.84, 1.38, 3.15, 3.18, 6.10 for CFS 2, 3, 4, 5 and 6–7(adjusted for age and sex) *30-day mortality* Frail patients: 50/190 (26.3%) Non-frail patients: 87/747 (11.6%) Unadjusted OR = 2.71 Compare to patients with CFS = 1, the adjusted ORs were 2.05, 3.11, 7.49, 9.79 and 10.40 for CFS 2, 3, 4, 5 and 6–7 (adjusted for age and sex) *Length of stay* Compare to patients with CFS = 1, the adjusted ORs were 1.21, 1.26, 1.48, 1.44 and 1.62 for CFS 2, 3, 4, 5 and 6–7
Carter et al. [29]	934	CFS	538 (58)	189 (20)	*Increased level of care* Frail patents: 101/189 (53.4%) Non-frail patients: 248/745 (33.3%) Unadjusted OR 2.30 Compare to patients with CFS = 1, the adjusted ORs were 2.14, 1.84, 4.48, 5.94 and 7.88 for CFS 2, 3, 4, 5 and 6–7 (adjusted for sex, age and care level before admission) *Length of stay* Compared to patients with CFS = 1, the adjusted HRs were 0.74, 0.66, 0.50, 0.52 and 0.55 for CFS 2, 3, 4, 5 and 6–7 (adjusted for sex, age and care level before admission)
Arteaga et al. [30]	92	CFS	49 (53)	23 (25)	*30-day mortality* Frail patients: 6/23 (26.1%) Non-frail patients: 4/69 (5.8%) Unadjusted OR = 2.71 *Complications* Frail patients: 9/23 (39.1%) Non-frail patients: 11/69 (15.9%) Unadjusted OR = 3.39
Vilches-Moraga et al. [31]	113	CFS	60 (53)	37 (33)	*1-year mortality* Frail patients: 22/37 (59.5%) Non-frail patients: 22/76 (28.9) Unadjusted OR 3.60 Compare to non-frail patients, frail patients had an adjusted HR of 5.40 (adjusted for ASA, reduced mobility and, peri-operative geriatric team)

*MFI* Modified Frailty Index, *OR* Odds Ratio, *CFS* Clinical Frailty Scale, *HR* Hazard Ratio

### Primary outcome

**Fig. 2:**
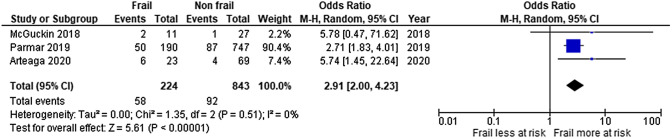
Forest plot for unadjusted OR of 30-day mortality in older patients undergoing emergency general surgery

Three studies reported 30-day mortality [26, 28, 30]. Based on the stratified data of the first one, we computed an unadjusted OR of 5.78 [26]. The second one reported an unadjusted OR of 2.71 [28]. There was also an increase in the adjusted OR with the increase in Clinical Frailty Scale (2.05, 3.11, 7.49, 9.79, and 10.40 for CFS 2, 3, 4, 5, and 6–7, respectively). The third, conducted in patients over 75, reported an unadjusted OR of 5.74[30]. The pooled OR, using random-effect models, was 2.91 (95% CI 2.00, 4.23). We did not observe any statistical heterogeneity between the studies. (Tau = 0.00, *I*^2^ = 0%) (Fig. 2). Based on the GRADE approach, the quality of this evidence is high (low risk of bias, large effect, and dose–response gradient).

### Secondary outcomes

One study reported 90-day mortality, with an unadjusted OR of 2.50 for frail patients compared to non-frail patients [28]. There was an increase in the adjusted OR with an increase in Clinical Frailty Scale (2.05, 3.11, 7.49, 9.79, and 10.40 for CFS 2, 3, 4, 5, and 6–7, respectively). One study reported 1-year mortality, with an unadjusted OR of 3.60 [31]. Two studies from the same cohort reported length of stay as an outcome [28, 29]. There was a significant association between frailty and length of stay (adjusted ORs were 1.21, 1.26, 1.48, 1.44, and 1.62 for CFS 2, 3, 4, 5, and 6–7).

Major complications (Clavien–Dindo ≥ 3) were reported in only one study [30]. There was a positive association between frailty and major post-operative complications, with an unadjusted OR of 3.39.

One study reported increased level of care as outcome and another study reported loss of functional independence at 1 year, defined as a Modified Barthel’s Index < 80/100 [27, 29]. Compared to non-frail patients, frailty was associated with both outcomes, with an unadjusted odds ratio 2.30 for increased level of care and of 4.42 for loss of functional independence at 1 year [27, 29]. For the increased level of care, the adjusted odds ratio was also progressively increasing for the different levels of frailty scores above 3 (4.48 for CFS 4, 5.94 for CFS 5, and 7.88 for CFS 6 or 7) [29].

### Quality assessment

Table 3 presents the quality assessment of the six studies, based on the Newcastle–Ottawa Scale, where ‘high’ quality choices are given a star from a minimum of 0 to a maximum of 9; more stars indicate less risk of bias and a higher study. Scores from the six studies ranged from 5 to 9. Exposed and non-exposed patients were from the same cohort and were representative of the community. In one study, the exposure was measured differently during the study (prospectively and retrospectively)[31]. Three studies did not present adjusted estimates [26, 27, 30]. The outcomes were mostly obtained by record linkage. Finally, the overall follow-up was judged as sufficient, with an important (31%) loss of follow-up for only one study [27].

**Table 3:** Results of the Newcastle–Ottawa Scale quality assessment

Authors	Year	Selection (4)	Comparability (2)	Outcome (3)
McGuckin et al. [26]	2018	****		***
Tan et al. [27]	2019	****		*
Parmar et al. [28]	2019	****	**	***
Carter et al. [29]	2020	****	**	***
Arteaga et al. [30]	2020	****		***
Vilches-Moraga et al. [31]	2020	***	*	***

## Discussion

This systematic review and meta-analysis found that frailty (measured by Clinical Frailty Scale ≥ 5) increased the odds of 30-day mortality in frail compared to non-frail older adults who underwent emergency general surgery. This systematic review also found increased odds of secondary outcomes including 90-day mortality, 1-year mortality, hospital length of stay, complications, and change in level of care at discharge using the Clinical Frailty Scale. There was evidence of increased loss of functional independence in frail patients (≥ 3/11) using the Modified Frailty Index. Several studies found increased odds of adverse outcomes for increasing scores on the Clinical Frailty Scale, consistent with dose–response using the Bradford Hill Criteria [32].

Several recent systematic reviews have assessed the impact of frailty on mortality in surgical patients [33–36]. Previous systematic reviews have found an association between frailty, mortality, and adverse functional outcomes after endovascular procedures for peripheral arterial disease, and in all vascular surgeries [33, 34]. Another recent systematic review in all surgical patients aged 60 years or older used the Fried frailty phenotype to categorize patients as frail vs not frail and robust vs pre-frail vs frail [35]. They found that the risk ratio (RR) of post-operative complications was 1.60 (1.20–2.13) when comparing frail patients to non-frail patients. Similarly, compared to the robust group, the risk ratio for complications was 1.77 (1.40–2.25) for the pre-frail group and 1.45 (1.17–1.80) for the frail group. Panayi et al. reported on the impact of frailty using the Modified Frailty Index on all surgical patients for post-operative complications, re-admission, re-operation, discharge to a skilled care facility, and mortality [36]. They included 16 studies in their meta-analysis and found that frail patients were more likely to experience complications (RR 1.48 [1.35–1.61]), major complications (RR 2.03 [1.26–3.29), wound complications (RR 1.52 [1.47–1.57]), re-admission (RR 1.61[1.44–1.80]), and discharge to skilled care (RR 2.15 [1.92–2.40]). In this study, the risk of mortality was also 4.19 ([2.96–5.92] *p* < 0.001) times higher in frail patients. However, emergency general surgery is relatively different from other surgeries, as mortality is often higher [4]. Our systematic review expands the understanding of the association between frailty and poor outcomes in the emergency general surgery population specifically.

The strengths of this systematic review are that this is the first the authors are aware of that pools’ results of the Clinical Frailty Scale to predict 30-day mortality in older adults undergoing emergency general surgery specifically. We used rigorous methodology according to PRISMA guidelines and had a strict age criterion for our included studies where all patients were age ≥ 65 years. This was evident in our low statistical heterogeneity. Therefore, the results of this study can be widely applied to emergency general surgery patients ≥ 65 years. Another strength is that five of the six included studies were prospective cohorts by design [27–31], four of which were considered low risk of bias according to the NOS scale.

This systematic review has several limitations. We only included studies that reported frailty measured by the Clinical Frailty Scale or the Modified Frailty Index. This decision was based on a preliminary literature search where studies we reviewed used these two tools most frequently; however, many of these studies were later excluded using other exclusion criteria. During the screening process, we identified several studies that could have been included, but used another tool to discriminate frail and non-frail patients. As we chose these two scores a priori, we continued our systematic review accordingly. Another limitation was the specific population; although many studies included patients over the age of 65 with emergency general surgery, they were often mixed with younger patients, patients without surgery, patients with non-emergency general surgery, or patients with different types of surgery (such as orthopaedic or vascular surgery). These studies were then excluded, because the proportion of emergency general surgery patients was very small or unknown. We attempted to mitigate this by contacting authors; however, we were not able to obtain stratified data for our specific population. Another limitation is that the meta-analysis was based on unadjusted estimates. The pooled estimate could therefore be biased due to confounding. Finally, our systematic review also only found one study meeting our eligibility criteria using the Modified Frailty Index.

Our study has several clinical and research implications. The first is that it can be widely applied to emergency general surgery patients ≥ 65 years of age as another tool to help patients and their families determine the patients’ risk of 30-day mortality based on their score on the Clinical Frailty Scale. For frail patients, they may choose a non-surgical option that is in keeping with their stated goals of care. On the other hand, older patients who score lower on the Clinical Frailty Scale may choose to pursue surgical interventions if it would improve their quality or quantity of life. It should be cautioned that the results of this study are not sufficient to promote the exclusive use of this scale to guide management decisions, as only two studies were included in the meta-analysis. However, the results of this meta-analysis do provide evidence that the Clinical Frailty Scale can be used as part of the decision-making process. The Clinical Frailty Scale can be widely, reliably, and rapidly applied by various healthcare providers in the acute care setting for geriatric patients at low cost [37]. Future research should investigate the use of the Clinical Frailty Scale with other risk factors for mortality to develop a more robust prognostic score for emergency general surgery patients ≥ 65 years. Additional meta-analyses are also required to compare different frailty scales in emergency general surgery patients ≥ 65 years.

## Conclusion

Frailty is significantly associated with worse outcomes after emergency general surgery in adults ≥ 65 years of age. The Clinical Frailty Scale could be used to improve preoperative risk assessment for patients and shared decision-making between patients and healthcare providers. Future research should explore the utility of the Clinical Frailty Scale in developing a prognostic score in emergency general surgery.

## Supplementary Information

Below is the link to the electronic supplementary material.

Supplementary file1 (DOCX 51 KB)

Supplementary file2 (DOCX 48 KB)

Supplementary file3 (PDF 131 KB)

## Data Availability

Data are available on request to the authors.
