# Supplementary material for: Association between mortality and frailty in emergency general surgery: a systematic review and meta-analysis
Source: Eur J Trauma Emerg Surg. 2021 Jan 9;48(1):141–51. doi: 10.1007/s00068-020-01578-9 (PMC8825621; doi:10.1007/s00068-020-01578-9)
Supplement: Supplementary file 2 — Supplementary file2 (DOCX 48 KB) [file 68_2020_1578_MOESM2_ESM.docx]

## Appendix II – Supplemental material

### S1 – Clinical Frailty Scale

| 1 – Very fit | People who are robust, active, energetic and motivated. These people commonly exercise regularly. They are among the fittest for their age. |
| --- | --- |
| 2 – Well | People who have no active disease symptoms but are less fit than category 1. Often, they exercise or are very active occasionally, e,g. seasonally. |
| 3 – Managing Well | People whose medical problems are well controlled, but are not regularly active beyond routine walking, |
| 4 – Vulnerable | While not dependent on others for daily help, often symptoms limit activities. A common complaint is being "slowed up", and/or being tired during the day. |
| 5 – Mildly Frail | These people often have more evident slowing, and need help in high order IADLs (finances, transportation, heavy housework, medications), Typically, mild frailty progressively impairs shopping and walking outside alone, meal preparation and housework. |
| 6 – Moderately Frail | People need help with all outside activities and with keeping house. Inside, they often have problems with stairs and need help with bathing and might need minimal assistance (cuing, standby) with dressing, |
| 7 – Severely Frail | Completely dependent for personal care, from whatever cause (physical or cognitive). Even so, they seem stable and not at high risk of dying (within - 6 months). |
| 8 – Very Severely Frail | Completely dependent, approaching the end of life. Typically, they could not recover even from a minor illness. |
| 9 – Terminally ill | Approaching the end of life. This category applies to people with a life expectancy <6 months, who are not otherwise evidently frail. |

### S2 – Modified Frailty Index

| History of diabetes mellitus |
| --- |
| History of congestive heart failure |
| History of hypertension requiring medication |
| History of either transient ischemic attack or cerebrovascular accident |
| Functional status 2 (not independent) |
| History of myocardial infarction |
| History of either peripheral vascular disease or rest pain |
| History of either chronic obstructive pulmonary disease or pneumonia |
| History of either prior percutaneous coronary intervention, prior cardiac surgery or angina |
| History of impaired sensorium |

### S3 – The Clavien-Dindo Classification

| Grade I | Any deviation from the normal postoperative course without the need for pharmacological treatment or surgical, endoscopic and radiological interventions Allowed therapeutic regimens are: drugs as antiemetics, antipyretics, analgesics, diuretics and electrolytes and physiotherapy. This grade also includes wound infections opened at the bedside. |
| --- | --- |
| Grade II | Requiring pharmacological treatment with drugs other than such allowed for grade I complications. Blood transfusions and total parenteral nutrition also included. |
| Grade III | Requiring surgical, endoscopic, or radiological intervention |
| *IIIa* | Intervention not under general anaesthesia |
| *IIIb* | Intervention under general anaesthesia |
| Grade IV | Life-threatening complication (including CNS complications) requiring IC/ICU-management |
| *IVa* | Single organ dysfunction (including dialysis) |
| *IVb* | Multiorgan dysfunction |
| Grade V | Death of a patient |
