# Supplementary material for: Association between mortality and frailty in emergency general surgery: a systematic review and meta-analysis
Source: Eur J Trauma Emerg Surg. 2021 Jan 9;48(1):141–51. doi: 10.1007/s00068-020-01578-9 (PMC8825621; doi:10.1007/s00068-020-01578-9)
Supplement: Supplementary file 3 — Supplementary file3 (PDF 131 KB) [file 68_2020_1578_MOESM3_ESM.pdf]

Appendix III – Search strategy

Database: Embase Classic+Embase <1947 to 2020 March 05>, Ovid MEDLINE(R) ALL <1946 to March 05, 2020>, EBM Reviews - Cochrane Central Register of Controlled Trials <January 2020>
Search Strategy:

----

1 Frail Elderly/ (21613)
2 Frailty/ (11653)
3 frail*.tw,kw. (57182)
4 1 or 2 or 3 (65667)
5 Emergencies/ or emergency treatment/ (114987)
6 Emergency Service, Hospital/ (72282)
7 emergenc*.tw,kw. (908260)
8 or/5-7 (959742)
9 exp Cholecystectomy/ (83048)
10 Laparotomy/ (103812)
11 exp Intestinal Obstruction/su (30149)
12 exp Peptic Ulcer/su (27498)
13 Tissue Adhesions/su (2251)
14 exp hernia/su (73921)
15 General Surgery/ (55360)
16 Colectomy/ (49075)
17 (general surg* or unplanned surg* or unschedul* surg*).tw,kw. (43872)
18 Appendectomy/ or appendicitis/su (37763)
19 or/9-18 (458952)
20 8 and 19 (30159)
21 ((emergenc* or urgent or expedit*) and (appendectom* or hernia* or colectom* or cholecystectom* or Laparotom* or bowel* or intestin* or ulcer* or surg* or obstruction* or rupture* or drainage*)).tw. (226027)
22 ((emergenc* or urgent or expedit*) and (appendectom* or hernia* or colectom* or cholecystectom* or Laparotom* or bowel* or intestin* or ulcer* or surg* or obstruction* or rupture* or drainage*)).kf. (1514)
23 20 or 21 or 22 (229948)
24 4 and 23 (852)
25 24 use medall (278) Medline
26 24 use cctr (31) Cochrane
27 frail elderly/ (21613)
28 frailty/ (11653)
29 frail*.tw. (54785)
30 27 or 28 or 29 (63739)
31 emergency treatment/ (28203)
32 emergency ward/ (208978)
33 emergency medicine/ or emergency care/ or emergency/ (232746)
34 emergenc*.tw. (896464)
35 or/31-34 (1030428)
36 exp cholecystectomy/ (83048)
37 laparotomy/ (103812)
38 exp gastrointestinal obstruction/su [Surgery] (16567)
39 exp peptic ulcer/su [Surgery] (27498)
40 tissue adhesion/su [Surgery] (2417)
41 exp hernia/su [Surgery] (73921)
42 general surgery/ (55360)
43 exp colon resection/ (44952)
44 (general surg* or unplanned surg* or unschedul* surg*).tw. (42243)
45 appendectomy/ (33404)
46 exp appendicitis/su [Surgery] (16468)
47 or/36-46 (441789)
48 35 and 47 (30428)

49 emergency surgery/ (25839)
50 ((emergenc* or urgent or expedit*) and (appendectom* or hernia* or colectom* or cholecystectom* or Laparotom* or bowel* or intestin* or ulcer* or surg* or obstruction* or rupture* or drainage*)).tw. (226027)
51 48 or 49 or 50 (241057)
52 30 and 51 (872)
53 52 use emczd (570) Embase
54 25 or 26 or 53 (879)
55 54 use medall (278)
56 54 use emczd (570) Embase
57 54 use cctr (31)

Scopus – 2020-03-06

(TITLE-ABS-KEY (surg*) OR TITLE-ABS-KEY (appendectom*) OR TITLE-ABS-KEY (hernia*) OR TITLE-ABS-KEY (colectom*) OR TITLE-ABS-KEY (cholecystectom*) OR TITLE-ABS-KEY (laparotom*) OR TITLE-ABS-KEY (obstruction*) OR TITLE-ABS-KEY (peptic AND ulcer*)) AND ((TITLE-ABS-KEY (emergenc*) OR TITLE-ABS-KEY (urgent) OR TITLE-ABS-KEY (expedited))) AND (TITLE-ABS-KEY (frail*))

Web of Science – 2020-03-06

# 5	336	#4 AND #3 *Indexes=SCI-EXPANDED, SSCI, A&HCI, CPCI-S, CPCI-SSH, ESCI Timespan=All years*	Edit	☐	☐
# 4	2,238,433	**TOPIC:** (surg*) *OR* **TOPIC:** (Cholecystectomy) *OR* **TOPIC:** (appendectom*) *OR* **TOPIC:** (hernia*) *OR* **TOPIC:** (obstruction*) *OR* **TOPIC:** (peptic ulcer*) *OR* **TOPIC:** (colectom*) *OR* **TOPIC:** (laparotom*) *OR* **TOPIC:** (drainage*) *OR* **TOPIC:** (rupture*) *Indexes=SCI-EXPANDED, SSCI, A&HCI, CPCI-S, CPCI-SSH, ESCI Timespan=All years*	Edit	☐	☐
# 3	1,359	#2 AND #1 *Indexes=SCI-EXPANDED, SSCI, A&HCI, CPCI-S, CPCI-SSH, ESCI Timespan=All years*	Edit	☐	☐
# 2	29,251	**TOPIC:** (frail*) *Indexes=SCI-EXPANDED, SSCI, A&HCI, CPCI-S, CPCI-SSH, ESCI Timespan=All years*	Edit	☐	☐
# 1	647,956	**TOPIC:** (emergenc*) *OR* **TOPIC:** (urgent) *OR* **TOPIC:** (expedited) *Indexes=SCI-EXPANDED, SSCI, A&HCI, CPCI-S, CPCI-SSH, ESCI Timespan=All years*			
